# A Novel Medical Treatment of Cushing's Due to Ectopic ACTH in a Patient With Neurofibromatosis Type 1

**DOI:** 10.5812/ijem.6898

**Published:** 2012-12-21

**Authors:** Gul Bano, Farheen Mir, Nigel Beharry, Philip Wilson, Shirley Hodgson, Stephen Schey

**Affiliations:** 1 Cellular and Molecular Medicine, St George’s University of London, London, UK; 2 Department of Paediatrics, Watford General Hospital, London, UK; 3 Department of Radiology, St. George's Health NHS Trust, London, UK; 4 Department of Cellular pathology, St. George's Healthcare NHS Trust, London, UK; 5 Clinical Developmental Sciences, St George’s University of London, London, UK; 6 Department of Haematology, Kings College London, SE5 9RS, London, UK

**Keywords:** Adrenal Gland Diseases, Adrenocortical Hyperfunction

## Abstract

A 64-year-old male presented with neurofibromatosis 1 and Cushing’s syndrome. Clinically he was over weight, depressed with extensive skin bruising and hypertension. His 24 hours urinary metanephrines, urinary 5HIAA, gut peptides and chromgranin levels were normal. His renal function and renal MRI scan was also normal. His cortisol failed to suppress on overnight dexamethsone suppression test. His low dose dexamethasone suppression with CRH stimulation showed failure of suppression of cortisol to < 50 nmol/L and ACTH was measurable at 10 ng/L on day 3. There was no response of ACTH or cortisol to CRH stimulation. His ACTH precursors were high at 126 pmol/L consistent with defective pro-opiomelanocortin (POMC) processing suggesting an ectopic source of ACTH production. The MRI scan of his pituitary and CT scan of the adrenal glands was normal. His octreotide scan was negative. The source of his ectopic ACTH was most likely a large retroperitoneal plexiform neurofibroma seen on CT abdomen that had undergone malignant peripheral nerve sheath tumour transformation on histology. He was a poor surgical risk for tumour debulking procedure. In view of the available literature and role of c-kit signalling in neurofibromatosis, he was treated with Imitinib. Four months after the treatment his Cushings had resolved on biochemical testing. After a year his plexiform neurofibroma has not increased in size. To our knowledge, this is the first case of NF1 associated with clinical and biochemical features of Cushing’s secondary to ectopic ACTH due to MPNST in a plexiform neurofibroma and its resolution on treatment with imatinib.

## 1. Introduction

Neurofibromas are the hallmark lesions of neurofibromatosis 1(NF1) and are benign nerve sheath tumours. The estimated incidence of NF1 is 1 in 3000. Approximately half of affected individuals represent first case as a result of a new genetic mutation (1). The NF1 gene is located on chromosome 17, encodes a protein named neurofibromin that works to control cellular proliferation through complex interactions with ras oncogenes (2). Patients with NF1 have lesions that are predominantly neuroectodermal or mesenchymal in origin.

Neurofibromas are categorized as localized, plexiform, or diffuse. Localized and plexiform neurofibromas of the paraspinal and sacral region are the most common abdominal neoplasm in NF1 (3).

Plexiform neurofibromas are variants of neurofibromas that involve a plexus of nerves or multiple fascicles within a medium- to large-sized nerve. They develop in about 40% of people with neurofibromatosis and occur exclusively in patients with NF1. The involved nerves retain their original configuration and anatomy but are altered into complex, tortuous masses. Plexiform neurofibromas are congenital lesions and tend to be larger, and more diffuse (4). These can transform to malignant peripheral nerve sheath tumour (MPNST). The paraspinal region of the abdomen, extremities, and head and neck region are the most common locations for MPNST (5). Tumours that arise in central locations such as the paraspinal region of the retroperitoneum are associated with lower 5-year survival rates, higher recurrence rates, and higher frequency of metastasis compared with tumours in other areas (6, 7).

Hypertension in NF1 can be seen at any age. Many adults with NF1 have usual essential form of hypertension. However, any person with NF1 and high blood pressure should be evaluated for two causes of hypertension mainly pheochromocytoma and vascular stenosis. Pheochromocytoma is more common in patients with NF1 compared with the general population, occurring in 0.1%–5.7% of patients with NF1 and in 20%–50% of NF1 patients with hypertension (8).

NF1 with optic pathway tumours (OPT) has been associated with of endocrine disorders such as growth hormone excess and precocious puberty (9).

NF1 can also be associated with a variety of endocrine tumours including somatostainoma, gastrointestinal stromal tumour (GIST) (10) and primary hyperparathyroidism (11).

We report a case of 64-year-old man who presented with clinical and biochemical features of Cushings due to ectopic ACTH syndrome in the setting of NF-1. The source of his ectopic ACTH secretion was most likely from the plexiform neurofibroma with MPNST. His treatment with Imatinib a tyrosine kinase inhibitor led to resolution of his Cushing's syndrome.

## 2. Case Report

**Figure 1.**
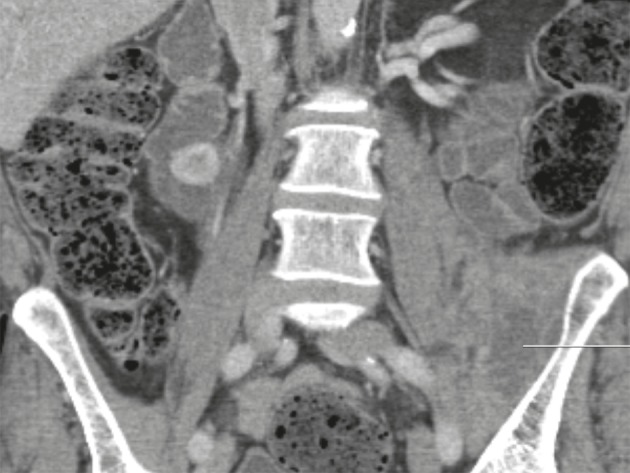
Mass Extending to Left Lesser Trochanter With Some Calcification

A 64 years old male with NF1 had multiple hospital admissions with abdominal and back pain between 2003 and 2004. He had lost significant amount of weight. As a part of work up he had MRI scan of the abdomen and pelvis. His scans showed a large pre-sacral mass in left retroperitoneal region with possible infiltrative characteristics. This mass extended up to left lesser trochanter and also had some calcification in it (Figure 1).

**Figure 2.**
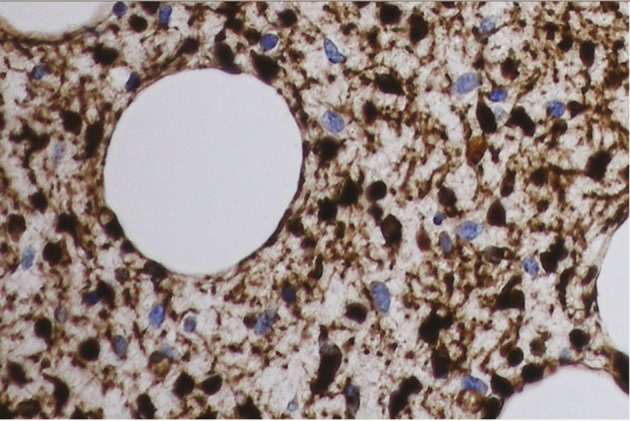
Spindle Cell Tumour Infiltrating Adjacent Fat and Entrapping Nerve Bundles. Tumour Cells Were Positive For s100 Protein Immunostains

**Figure 3.**
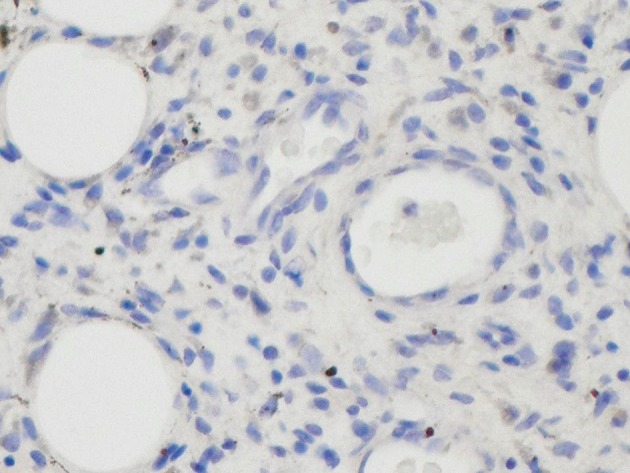
Immunochemical Staining of The Tumour Did Not Show Positive Staining for Acth

A diagnosis of plexiform neurofibroma was made. In 2008 he had acute abdominal pain following trauma. There was possibility of a haematoma in the retroperitoneal mass on imaging. He had surgery with drainage of haematoma and debulking of retroperitoneal mass. He bled heavily during the surgery and required blood transfusion. The mass removed surgically showed diffuse involvement of nerve segment and its branches with tortuous expansion. Histology revealed spindle cell tumour infiltrating adjacent fat and entrapping nerve bundles (Figures 2 and 3). Tumour cells were positive for S100 protein immunostains. These features were consistent with malignant peripheral nerve sheath tumours. He was reviewed in genetic endocrine clinic. He appeared depressed and was overweight with a BMI of 29kg/m2. He had extensive bruising of skin on his arms and legs and his blood pressure was 160/100mmHg. He had no abdominal striae or proximal muscle weakness. His 24 hours urinary metanephrines and 5 HIAA were normal. His serum potassium, renal function and renal MRI scan was normal. He had a 2 cm enhancing mass in the wall of the second part of the duodenum representing neurofibroma. His gut peptides, chromgranin A and B were normal. His octreotide scan was negative.

He had overnight dexamethasone suppression test and his cortisol failed to suppress. He had low dose dexamethasone suppression test (LDDST) combined with CRH stimulation. At baselne he had measurable ACTH of 29ng/L with cortsiol of 689nmol/L. His cortisol was 427nmol/L on day three of low dose dexamethasone and his ACTH was 10ng/L consistent with ACTH dependent Cushing’s. After 100mcg of CRH there was no increase in cortisol or ACTH level. His ACTH precursors were high at 126 pmol/L (Table 1). A CT scan of his adrenals and MRI scan of the pituitary gland was normal. Patient did not have inferior petrosal sinus sampling in view of normal pituitary MRI scan, multiple neurofibromas and previous history of bleeding on surgery.

A diagnosis of Cushing’s syndrome due to ectopic ACTH was made. The source of ectopic ACTH was most likely his large retroperitoneal plexiform neurofibroma with MPNST. This patient was a poor surgical risk for further debulking of the plexifrom neurofibroma. He was treated with Imatinib mesylate at a daily dose of 75 mg/kg for 4 weeks.

He had repeat LDDST with CRH stimulation 4 months after the treatment. His cortisol suppressed to 27 nmol/L and ACTH was < 5ng/L on day 3. There was no response of cortisol or ACTH to CRH (Table 2). His MRI scan of the abdomen one year after treatment with imitinib showed no increase in the size of plexiform neurofibroma but there is a definite decrease in the density of plexiform mass. Immunochemical staining of the tumour did not show positive staining for ACTH.

**Table 1:** Low Dose Dexamethasone Suppression Test Combined with CRH Stimulation

Day	Time	Cortisol, nmol/L	ACTH, ng/L
**1**	0	689	29
**3**	0	427	10
** **	15	401	13
** **	30	403	15

No suppression of cosrtisol on day 3 after low dose dexamethasone and no increase in cortisol or ACTH after CRH stimulation

ACTH:35ng/L

ACTH precursors:126pmol/L

**Table 2:** Low Dose Dexamethasone SuppressionTest Combined With CRH Stimulation After Imitinib Therapy

Day	Time	Cortisol a, nmol/L	ACTH, ng/L
**1**	0	691	67
**3**	0	23	< 5
	15	24	< 5
	30	29	< 5

^a^ Suppression of cosrtisol on day 3 after low dose dexamethasone with suppressed ACTH and no increase in cortisol or ACTH after CRH stimulation indicates normal response

## 3. Discussion

Neurofibromatosis evolves from mutations in the *NF1* tumor suppressor gene. It leads to recruitment of mast cells into neurons via *c-kit* signalling. The mast cells then initiate matrix formation and angiogenesis required for neurofibroma development (12). The natural history of plexiform neurofibromas has not been clearly elucidated but they do have potential for malignant transformation to malignant peripheral nerve sheath tumor (MPNST). MPNSTs are not uncommon in adolescents and adults with NF1. The lifetime risk of developing an MPNST for a person with *NF1* is 4%–5% (13). MPNST is an aggressive malignancy and can be fatal due to organ invasion or malignant transformation. These tumors do not respond to chemotherapy and can be difficult if not impossible to remove surgically. MPNSTs in patients with NF1 carry a poorer prognosis than in patients without this condition and tumor volume is an independent prognostic indicator (14).

The Ectopic ACTH syndrome (EAS) occurs in around 5–10% of all cases of ACTH-dependent hypercortisolism (15).

Ectopic ACTH- and CRH-secreting tumors arise from nonpituitary neoplasms, and may be clinically overt or occult. Ectopic ACTH secretion Cushing's syndrome is associated with significant morbidity and mortality. Management of this Cushing’s requires control of the hypercortisolaemia as well as identification and treatment of the underlying source adrenocorticotrophin. ACTH is derived by cleavage from the precursor, pro-opiomelanocortin (POMC). In human pituitary POMC is enzymatically processed to pro-ACTH, which is then cleaved to give ACTH (16). Ectopic 'ACTH' is characterized by aberrant processing of POMC. POMC expressing cells can potentially secrete number of ACTH related peptides depending on the degree of processing by the tissue or tumor. The precursors of ACTH (POMC and proACTH) in the circulation of normal subjects are in the range of 5-40 pmol/L (17). Pituitary tumors have low levels of ACTH precursors (below 100 pmol/L) and the mean ratio of ACTH precursors to ACTH is 5:1. In ectopic Cushing's Syndrome,the extra pituitary tumour cells are unable to process POMC efficiently. Therefore there is an increased level of ACTH precursors in the circulation in patients with the ectopic ACTH Syndrome. The diagnostic accuracy of the measurement of ACTH precursors has been compared with the current "gold standard" test of inferior petrosal sinus sampling (IPSS). All patients with ACTH precursors below a diagnostic cut-off of 100 pmol/L were subsequently shown to have pituitary tumors, whereas levels of >100 pmol/L were seen in patients with ectopic ACTH producing tumors. Therefore measurement of ACTH precursors offers a simple non-invasive diagnostic test for the differential diagnosis of Cushing's syndrome and compares favourably with IPSS (18).

Patients with *NF1* are at an approximately 2–4 fold higher risk of developing tumors than the general population with a risk of malignancy estimated at between 5 and 15 percent (19, 20). The gastrointestinal tract may be involved in *NF-1* and includes mucosal and myenteric nerve hyperplasia, gastrointestinal stromal tumors (GIST), carcinoids, pheochromocytomas, paragangliomas, as well as pancreatic neuroendocrine tumors (21).

Neurofibromin is a tumor suppressor gene and mutations in *NF-1* cause increased activation of ras and increased signaling via downstream pathways, including the mitogen-activated protein kinase (MAPK) pathway and the mammalian target of rapamycin (mTOR) pathway. mTOR is a member of the phosphoinositide-3-kinase-related family, and serves as a critical mediator of cell growth and proliferation, responsible for stimulating cell growth and controlling cellular energy levels. Pathogenic mutations of neurofibromin lead to constitutive activation of downstream pathways and subsequent tumor development (22).

Imatinib inhibits c-kit signalling. It is an approved treatment for chronic myeloid leukemia and certain stomach and digestive system tumours (23, 24). Imatinib mesylate (Glivec) is known to inhibit Schwann cell viability and to reduce the size of human Plexiform neurofibroma in a xenograft model.The discovery that *c-kit* plays a central role in neurofibromatosis evolution led the investigators to use Imatinib (25).

Initial compassionate use of the drug in a three-year-old girl with the NF1 resulted in about 70% shrinkage of a plexiform neurofibroma that was causing life-threatening airway compression (26). There is an ongoing Pilot Study to determine the efficacy of imitanib in NF1 patients with plexiform neurofibromas using new response assessment modalities with the secondary goals of assessing imitanib toxicity, and characterizing markers of response. Imtinib is being used in different doses starting from 400mg to 800mg a day. The treatment will be continued for 6 to 24 if the patient is deriving a clinical benefit (27). Our case of NF1 presented with Cushings due to ectopic ACTH. The source of ectopic ACTH was thought to be his large retroperitoneal plexiform neurofibroma with MPNST. The ACTH staining for the tumour removed surgical was negative.

As this patient was a poor surgical risk for further debulking of the plexifrom neurofibroma, we treated him with Imatinib mesylate at a daily dose of 75 mg/kg for 4 weeks. He had no side effects from the drug. His repeat tests 4 months after the treatment suggest that his Cushings has resolved. His plexiform neurofibroma has remained stable. His mass probably would shrink if we used imatninb for longer period of time. To our knowledge this is the first case report of Cushing’s due to an Ectopic ACTH associated with NF1 and its treatment with imatinib in an adult.
